# Phylogeography and Population Structure Analysis Reveal Diversity by Gene Flow and Mutation in *Ustilago segetum* (Pers.) Roussel *tritici* Causing Loose Smut of Wheat

**DOI:** 10.3389/fmicb.2019.01072

**Published:** 2019-05-15

**Authors:** Prem Lal Kashyap, Sudheer Kumar, Rahul Tripathi, Ravi Shekhar Kumar, Poonam Jasrotia, Devendra Pal Singh, Gyanendra Pratap Singh

**Affiliations:** ICAR-Indian Institute of Wheat and Barley Research (IIWBR), Karnal, India

**Keywords:** genetic diversity, gene flow, haplotype, mutation, population structure

## Abstract

*Ustilago segetum* (Pers.) Roussel *tritici* (UST) causes loose smut of wheat account for considerable grain yield losses globally. For effective management, knowledge of its genetic variability and population structure is a prerequisite. In this study, UST isolates sampled from four different wheat growing zones of India were analyzed using the second largest subunit of the RNA polymerase II (*RPB2*) and a set of sixteen neutral simple sequence repeats (SSRs) markers. Among the 112 UST isolates genotyped, 98 haplotypes were identified. All the isolates were categorized into two groups (*K* = 2), each consisting of isolates from different sampling sites, on the basis of unweighted paired-grouping method with arithmetic averages (UPGMA) and the Bayesian analysis of population structure. The positive and significant index of association (I_A_ = 1.169) and standardized index of association (rBar_D_ = 0.075) indicate population is of non-random mating type. Analysis of molecular variance showed that the highest variance component is among isolates (91%), with significantly low genetic differentiation variation among regions (8%) (F_st_ = 0.012). Recombination (R_m_ = 0) was not detected. The results showed that UST isolates have a clonal genetic structure with limited genetic differentiation and human arbitrated gene flow and mutations are the prime evolutionary processes determining its genetic structure. These findings will be helpful in devising management strategy especially for selection and breeding of resistant wheat cultivars.

## Introduction

Loose smut caused by the basidiomycete fungus *Ustilago segetum* (Pers.) Roussel *tritici* Jensen (UST), is one of the most serious diseases on wheat (*Triticum aestivum* L.) globally. The disease is favored by moist and cool climate during anthesis (Quijano et al., 2016). This fungus converts the spike floral tissues to fungal teliospores, causing yield losses equivalent to the percent smutted spikes (Green et al., 1968; Singh, 2018). The primary inoculum of the pathogen survives in the embryo of the wheat seeds (Kassa et al., 2015). Wilcoxon and Saari (1996) documented that the fungus can result in reductions of 5–20 per cent profit at an infection level of 1–2 per cent. Similarly, Nielsen and Thomas (1996) reported 15–30% annual yield losses as a result of UST infection. Joshi et al. (1980) reported loose smut incidence up to 10% in North Western parts of India. Besides India, 5–10% loose smut incidence was also reported from Russia, New Zealand, and USA (Thomas, 1925; Bonne, 1941; Atkins et al., 1943; Watts Padwick, 1948; Menzies et al., 2009; Kaur et al., 2014).

The infection process and disease cycle of UST on wheat has been elaborately discussed by several workers (Wilcoxon and Saari, 1996; Ram and Singh, 2004). Dikaryotic spores of UST disembarked on the wheat floret, germinate and penetrate the ovary through feathery stigma during anthesis (Dean, 1964; Shinohara, 1976). Mycelia of UST stay alive within the embryo of infected seeds and move systemically through the growing point of the tillers without showing any visible symptoms (Kumar et al., 2018). The symptoms become visible on emergence of spikes from the boot. Several methods are available to manage loose smut that include use of disease free seed, seed treatment with hot water or systemic fungicides, and host resistance are highly effective in controlling loose smut of wheat (Jones, 1999; Bailey et al., 2003; Knox et al., 2014). Unfortunately, the high genetic variability in the pathogen population may develop strains resistant to fungicides and also reduces lifespan of the resistant varieties (Randhawa et al., 2009). Therefore, the understanding of the variability and mechanism causing variability in the pathogen population is important for framing effective disease management and resistance breeding strategies.

Traditionally, variations in fungal pathogens have been deciphered on the basis of morphology, cultural characters, physicochemical characters, virulence pattern, mating type, and disease reaction on differential hosts (Kaur et al., 2014; Kashyap et al., 2015; Yu et al., 2016). Unfortunately, these methods are time consuming, highly influenced by environment and thus are not very precise. Recently, DNA profiling based on restriction fragment length polymorphism (RFLP), random amplified polymorphic DNA (RAPD), amplified fragment length polymorphism (AFLP) and inter simple sequence repeat (ISSR) are being extensively employed to study the population biology and genetic diversity among fungi (Bennett et al., 2005; Kashyap et al., 2016; Kumar et al., 2016; Yu et al., 2016). Karwasra et al. (2002) used RAPD, ISSR, and AFLP profiling for assessing the extent of genetic variation among the regional UST isolates collected from Haryana, India. SSR or microsatellites markers have an advantage in studying genetic diversity, population genetic structure, genetic linkage mapping, and quantitative trait locus (QTL) because of its high repeatability, transferability, co-dominance, and ubiquitous presence (Ellegren, 2004; Kumar et al., 2013a; Singh et al., 2014; Zhu et al., 2016). Recently, SSR has been used for deciphering diversity in many fungal plant pathogens, such as *Puccinia triticina* (Wang et al., 2009), *Magnaporthe grisea* (Shen et al., 2004), *Rhynchosporium secalis* (Bouajila et al., 2007), *Phytophthora infestans* (Zhao et al., 2008), *Phaeosphaeria nodorum* (Sommerhalder et al., 2010), *Fusarium culmorum* (Pouzeshimiab et al., 2014), *Ustilago hordei* (Yu et al., 2016), *Puccinia graminis* f. sp. *tritici* (Prasad et al., 2018), and *Bipolaris oryzae* (Ahmadpour et al., 2018). Storch et al. (2007) reported that protein-coded genes are generally more conserved and can be aligned with more reliability. Among protein-coding markers, second-largest subunit of nuclear RNA polymerase II (*RPB2*), translation elongation factor 1-alpha (Tef1), beta-tubulin (Tub2) and actin (ACT) have been most frequently used for inferring phylogenetic relationships among fungi (Stielow et al., 2015; Raja et al., 2017). Functionally, *RPB2* gene is responsible for the transcription of protein-encoding genes (Sawadogo and Sentenac, 1990) and present as single-copy in all eukaryotes (Thuriaux and Sentenac, 1992). A high level of polymorphisms in this gene makes this an excellent tool to study molecular evolution and phylogenetic relationships (Matheny et al., 2007; Krimitzas et al., 2013; Wang et al., 2016; Kruse et al., 2017). Stockinger et al. (2014) reported *RPB2* gene as a potential marker for adequate phylogenetic resolution to resolve fungal lineages when compared to rDNA loci. Therefore, in the present study phylogenetic analysis of the single copy of *RPB2* gene was done to explore genetic differentiation of UST populations. Despite recent advances, the role of gene flow in the reproduction, dispersal and evolution of UST populations is still poorly studied. To the best of our knowledge, fingerprinting and genetic diversity in UST population using microsatellite markers have not been studied extensively on large number of UST isolates. Thus, the present investigation was undertaken to study the genetic variation in the UST isolates collected from four different agro-ecological zones of India. The specific objectives of present investigation were to: (i) analyze the genetic diversity of UST isolates of Indian origin based on geographic areas of collection by microsatellites and *RPB2* gene sequence comparison (ii) investigate the possibility of random mating within their sampling sites using MULTILOCUS version 1.31 (Agapow and Burt, 2001) and (iii) determine the population genetic structure of the UST population in four different wheat growing zones by employing genetic data analysis tools like GenAlEx 6.5 (Peakall and Smouse, 2012), NTSYS-pc program V2.1 (Rohlf, 2002), DnaSP program (Tajima, 1983), STRUCTURE 2.3.4 (Pritchard et al., 2000), and Bottleneck v1.2 (Agapow and Burt, 2001).

## Materials and Methods

### Sampling, Isolation and Purification of Fungal Isolates

One hundred and twelve isolates of UST were collected from different agro-ecological zones of India during 2016–2018 (Table 1, Figure S1). Stratified random sampling method was adopted at spike emergence to anthesis stage at least 30 KM apart, in each field. One smutted spike was gathered per field to avoid the possibility of mixture of genotypes. Isolates were assigned into four populations and named as Central zone (CZ), North Eastern Plain Zone (NEPZ), North Hill Zone (NHZ), and North Western Plain Zone (NWPZ). Single-teliospore cultures were obtained by inoculating teliospores on half-strength potato dextrose agar (50% PDA) and incubated at 25 ± 1°C for 24 h. Single germinating teliospores were transferred to slants containing 50% PDA, incubated at 25 ± 1°C and maintained at 4°C for further use.

**Table 1:** Sampling details of *Ustilago segetum tritici* (UST) isolates used in the study.

**S.N**.	**Isolate code**	**Region/State**	**Zone**	**Latitude (*N*)**	**Longitude (*E*)**	**UTM (*E*)**	**UTM (*N*)**	**HG**	**HC**	**NCBI accession**
1	WLS17-PUN-1	Yodhapur, Punjab	NWPZ	30.084	76.573	651593.80	3329136.60	52	1	MH160206
2	WLS17-PUN-2	Mirapur, Punjab	NWPZ	30.200	76.492	643618.00	3341932.40	1	2	MH160207
3	WLS17-HR-3	Taprapur, Haryana	NWPZ	30.267	77.152	707042.00	3350276.80	53	3	MH160208
4	WLS17-HR-4	Gagsina, Haryana	NWPZ	29.568	76.886	682707.80	3272403.20	54	1	MH160209
5	WLS17-HR-5	Samora, Haryana	NWPZ	29.816	77.006	693861.70	3300051.80	55	3	MH160210
6	WLS17-HR-6	Indri, Haryana	NWPZ	29.951	77.048	697648.50	3315152.90	56	1	MH160211
7	WLS17-HR-7	Stondi, Haryana	NWPZ	29.570	76.895	683555.80	3272583.30	57	1	MH160212
8	WLS15-WB-8	Karimpur, West Bengal	NEPZ	23.545	88.539	657067.90	2604691.20	12	1	MH160213
9	WLS17-HR-9	Gagsina, Haryana	NWPZ	29.568	76.886	682707.80	3272403.20	58	1	MH160214
10	WLS16-WB-10	Muradpur (Jalangi), West Bengal	NEPZ	24.089	88.696	672410.80	2665131.90	13	1	MH160215
11	WLS15-WB-11	Isalampur, West Bengal	NEPZ	26.227	88.146	614505.40	2901327.10	14	2	MH160216
12	WLS15-WB-12	Dakashin Dinapur, West Bengal	NEPZ	25.264	88.905	691837.20	2795509.90	15	1	MH160217
13	WLS17-HR-13	Budanpur, Haryana	NWPZ	29.593	76.914	685393.00	3275245.30	14	2	MH160218
14	WLS17-MH-14	Pune, Maharashtra	CZ	18.483	73.779	371125.30	2044019.40	1	2	MH160219
15	WLS17-MH-15	Wakapur, Maharashtra	CZ	20.726	76.967	704857.90	2293078.00	2	1	MH160220
16	WLS17-HR-16	Mohay-Ud-Dinpur, Haryana	NWPZ	29.644	77.076	700955.90	3281138.80	59	1	HD 2967
17	WLS17-HR-17	Bati, Haryana	NWPZ	29.697	76.297	625522.10	3285898.80	60	1	HD 2967
18	WLS17-PUN-18	Langroya, Punjab	NWPZ	31.111	76.174	611948.10	3442538.30	61	1	MH160223
19	WLS17-MH-19	Washim, Maharashtra	CZ	20.195	76.925	701191.80	2234174.80	3	1	MH160224
20	WLS17-HR-20	Laha, Naraingarh, Haryana	NWPZ	30.313	77.066	698615.60	3355265.50	24	5	MH160225
21	WLS17-HR-21	Dhanoli, Ambala, Haryana	NWPZ	30.232	77.054	697671.80	3346277.90	62	1	MH160226
22	WLS17-HR-22	Pathreri, Haryana	NWPZ	30.254	77.016	693922.80	3348650.20	53	3	MH160227
23	WLS17-HR-23	Pratapgarh, Kurukhestra, Haryana	NWPZ	29.982	76.880	681336.50	3318261.00	63	1	MH160228
24	WLS17-HR-24	Ladwa, Haryana	NWPZ	29.991	77.038	696558.40	3319515.30	64	1	MH160229
25	WLS17-UP-25	Sherkot, Uttar Pradesh	NWPZ	29.322	78.593	266235.60	3246067.60	55	3	MH160230
26	WLS17-RJ-26	Badh Fatehpura, Rajasthan	NWPZ	26.888	75.559	555511.30	2974114.30	65	1	MH160231
27	WLS17-RJ-27	Ramkui, Rajasthan	NWPZ	26.960	75.550	554569.00	2982086.90	66	1	MH160232
28	WLS17-RJ-28	Sanganer, Rajasthan	NWPZ	26.819	75.696	569125.00	2966539.20	67	1	MH160233
29	WLS17-RJ-29	Boraj, Rajasthan	NWPZ	26.862	75.448	544455.90	2971262.00	68	1	MH160234
30	WLS17-UK-30	Domet, Uttrakhand	NHZ	30.511	77.854	773937.40	3378919.90	24	5	MH160235
31	WLS17-UP-31	Unnao, Uttar Pradesh	NWPZ	26.536	80.486	448834.20	2935181.00	69	1	MH160236
32	WLS17-UP-32	Gazipur, Uttar Pradesh	NWPZ	25.788	83.171	717682.80	2854017.20	70	1	MH160237
33	WLS17-MH-33	Brahmangaon, Maharashtra	CZ	20.552	74.302	427242.10	2272723.10	4	2	MH160238
34	WLS17-UP-34	Nethaur, Uttar Pradesh	NWPZ	29.327	78.392	246715.10	3246986.20	55	3	MH160239
35	WLS17-UP-35	Shahadatpura, Uttar Pradesh	NWPZ	25.935	83.569	757294.60	2870954.70	71	1	MH160240
36	WLS17-UP-36	Shahganj, Uttar Pradesh	NWPZ	26.051	82.678	667882.60	2882380.80	72	1	MH160241
37	WLS17-UP-37	Jaunpur, Uttar Pradesh	NWPZ	25.743	82.702	670726.90	2848327.10	73	1	MH160242
38	WLS17-MH-38	Baramati, Maharashtra	CZ	18.139	74.574	454965.60	2005623.70	5	1	MH160243
39	WLS17-UK-39	Almora, Uttrakhand	NHZ	29.791	73.536	358481.80	3296537.70	25	1	MH160244
40	WLS17-UK-40	Kaladungi, Uttrakhand	NHZ	29.295	79.336	338358.60	3241810.20	26	1	MH160245
41	WLS17-UK-41	Quano, Uttrakhand	NHZ	30.677	77.764	764845.00	3397092.30	4	2	MH160246
42	WLS17-MH-42	Jopul, Maharashtra	CZ	20.190	73.933	388515.80	2232865.20	6	1	MH160247
43	WLS17-PUN-43	Fatehgarh Sahib, Punjab	NWPZ	31.509	76.223	616103.60	3486677.80	74	1	MH160248
44	WLS17-PUN-44	Ropar, Punjab	NWPZ	31.246	76.473	640300.80	3457750.60	75	1	MH160249
45	WLS17-PUN-45	Bhalowal, Punjab	NWPZ	31.318	76.423	635408.90	3465683.00	76	1	MH160250
46	WLS16-WB-46	Petrapole, West Bengal	NWPZ	23.036	88.877	692320.50	2548732.70	16	1	MH160251
47	WLS17-PUN-47	Hiyatpur, Punjab	NWPZ	31.145	76.224	616681.90	3446297.40	77	1	MH160252
48	WLS17-PUN-48	Hakimrpur Apra, Punjab	NWPZ	31.106	75.907	586525.20	3441703.60	78	1	MH160253
49	WLS17-PUN-49	Kalpi, Punjab	NWPZ	30.288	77.000	692344.90	3352378.30	79	1	MH160254
50	WLS17-PUN-50	Rupnagar, Punjab	NWPZ	30.991	76.532	646292.50	3429639.10	80	1	MH160255
51	WLS17-PUN-51	Sherpur Gill, Punjab	NWPZ	31.082	76.265	620635.20	3439429.50	81	1	MH160256
52	WLS17-PUN-52	Sibalmajra, Punjab	NWPZ	31.099	76.242	618492.20	3441213.70	82	1	MH160257
53	WLS17-PUN-53	Gurdashpur, Punjab	NWPZ	32.046	75.383	536162.00	3545619.30	83	1	MH160258
54	WLS17-PUN-54	Moga, Punjab	NWPZ	30.837	75.158	515088.80	3411540.20	84	1	MH160259
55	WLS17-PUN-55	Khabra, Punjab	NWPZ	31.228	76.110	605691.40	3455353.50	85	1	MH160260
56	WLS17-PUN-56	Dasuya, Punjab	NWPZ	31.813	75.656	562100.00	3519909.40	86	1	MH160261
57	WLS17-PUN-57	Jalandar, Punjab,	NWPZ	31.298	75.544	551727.20	3462736.10	87	1	MH160262
58	WLS17-PUN-58	Ludhiana, Punjab	NWPZ	30.906	75.806	576977.70	3419451.90	88	1	MH160263
59	WLS17-JK-59	Kathua, Jammu and Kashmir	NHZ	32.426	75.437	541057.80	3587777.80	27	1	MH160264
60	WLS17-JK-60	RS Pura, Jammu and Kashmir	NHZ	32.736	74.830	484087.30	3622074.90	28	1	MH160265
61	WLS17-JK-61	Chatha, Jammu and Kashmir	NHZ	32.640	74.800	481229.90	3611389.90	29	1	MH160266
62	WLS17-JK-62	Barnai, Jammu and Kashmir	NHZ	32.709	74.789	480216.50	3619064.90	30	1	MH160267
63	WLS17-JK-63	Udhaywall, Jammu and Kashmir	NHZ	32.741	74.821	483266.30	3622559.80	31	1	MH160268
64	WLS17-JK-64	Ramghar, Jammu and Kashmir	NHZ	32.554	74.900	490617.60	3601873.00	32	1	MH160269
65	WLS17-JK-65	Kana check, Jammu and Kashmir	NHZ	32.757	74.849	485811.80	3624358.70	33	1	MH160270
66	WLS17-JK-66	Domana, Jammu and Kashmir	NHZ	32.769	74.817	482841.90	3625643.60	34	1	MH160271
67	WLS17-JK-67	Ranbir Singh pura, Jammu and Kashmir	NHZ	32.606	74.734	475034.80	3607688.00	35	1	MH160272
68	WLS17-PUN-68	Kurdan, Phillaur Punjab	NWPZ	31.047	75.944	590070.80	3435193.70	89	1	MH160273
69	WLS17-PUN-69	Khadukhera, Punjab	NWPZ	30.563	76.464	640377.70	3382102.30	43	2	MH160274
70	WLS17-MP-70	Indore, Madhya Pradesh	CZ	22.698	75.880	590380.70	2510394.30	7	1	MH160275
71	WLS17-MP-71	Ujjain, Madhya Pradesh	CZ	23.214	75.786	580451.80	2567464.00	8	1	MH160276
72	WLS17-MP-72	Rewa, Madhya Pradesh	CZ	24.541	81.302	530568.70	2714113.90	9	1	MH160277
73	WLS17-MP-73	Bhopal, Madhya Pradesh	CZ	23.256	77.410	746609.30	2573922.20	10	1	MH160278
74	WLS17-MP-74	Arjani, Madhya Pradesh	CZ	23.820	78.717	267429.30	2636195.10	11	1	MH160279
75	WLS17-RJ-75	Rajpura, Rajasthan	NWPZ	26.795	76.002	599620.30	2964152.30	90	1	MH160280
76	WLS17-RJ-76	Goner, Rajasthan	NWPZ	26.823	75.930	592427.20	2967202.70	91	1	MH160281
77	WLS17-HP-77	Bara, Himachal Pradesh	NHZ	31.481	76.575	649577.80	3484017.90	36	1	MH160282
78	WLS17-HP-78	Rudhanni, Himachal Pradesh	NHZ	31.453	76.690	660539.10	3481039.00	24	5	MH160283
79	WLS17-HP-79	Kangra, Himachal Pradesh	NHZ	32.109	76.266	619457.20	3553261.30	37	1	MH160284
80	WLS17-HP-80	Mandi, Himachal Pradesh	NHZ	31.680	76.940	683934.70	3506583.40	38	1	MH160285
81	WLS17-HP-81	Ratti, Himachal Pradesh	NHZ	31.604	76.903	680558.50	3498104.00	39	1	MH160286
82	WLS17-HP-82	Ramshehar, Himachal Pradesh	NHZ	31.091	76.790	670693.40	3441100.20	40	1	MH160287
83	WLS17-HP-83	Una, Himachal Pradesh	NHZ	31.472	76.243	618040.90	3482586.40	41	1	MH160288
84	WLS17-HP-84	Sundar Nagar, Himachal Pradesh	NHZ	31.171	76.853	676644.10	3450015.20	42	1	MH160289
85	WLS17-HP-85	Kullu, Himachal Pradesh	NHZ	31.954	77.095	698035.70	3537234.10	43	2	MH160290
86	WLS17-UP-86	Shamli, Uttar Pradesh	NWPZ	29.625	77.090	702362.60	3279039.20	92	1	MH160291
87	WLS17-UP-87	Mohamdabad, Uttar Pradesh	NWPZ	28.772	78.164	223090.50	3186033.70	24	5	MH160292
88	WLS17-UP-88	Rajak Nagar, Uttar Pradesh	NWPZ	29.495	77.245	717686.80	3264898.00	53	3	MH160293
89	WLS17-UP-89	Faizabad, Uttar Pradesh	NWPZ	26.769	82.145	613856.10	2961394.30	93	1	MH160294
90	WLS17-HP-90	Kunihar, Himachal Pradesh	NHZ	31.084	76.969	687785.90	3440527.40	44	1	MH160295
91	WLS17-HP-91	Diggal, Himachal Pradesh	NHZ	31.084	76.869	678280.10	3440428.70	45	1	MH160296
92	WLS17-HP-92	Jai Nagar, Himachal Pradesh	NHZ	31.171	76.853	676644.10	3450015.20	46	1	MH160297
93	WLS17-JK-93	Khandwalmore, Jammu and Kashmir	NHZ	32.405	75.307	528843.30	3585363.40	47	2	MH160298
94	WLS17-JK-94	Dablehar, Jammu and Kashmir	NHZ	32.576	74.760	477448.10	3604322.60	48	2	MH160299
95	WLS17-JK-95	Satwari, Jammu and Kashmir	NHZ	32.737	74.831	484195.20	3622165.00	24	5	MH160300
96	WLS17-JK-96	Saikalan, Jammu and Kashmir	NHZ	32.507	74.791	480370.00	3596643.80	49	1	MH160301
97	WLS17-JK-97	Udhaywalla, Jammu and Kashmir	NHZ	32.741	74.821	483266.30	3622559.80	48	2	MH160302
98	WLS17-JK-98	Quderpur, Jammu and Kashmir	NHZ	32.578	74.760	477448.50	3604544.40	47	2	MH160303
99	WLS17-JHK-99	Dhampur, Jharkhand	NEPZ	23.921	86.134	411867.50	2645781.20	17	1	MH160304
100	WLS17-JHK-100	Kannu, Jharkhand	NEPZ	24.247	87.255	525877.30	2681561.20	18	1	MH160305
101	WLS17-JHK-101	Jharkhandi, Jharkhand	NEPZ	24.344	86.026	401160.80	2692643.80	19	1	MH160306
102	WLS17-JHK-102	Deoghar, Jharkhand	NEPZ	24.471	86.693	468928.00	2706386.60	20	1	MH160307
103	WLS17-JHK-103	Gola-Baniyatu, Jharkhand	NEPZ	23.498	85.660	363208.90	2599294.30	21	1	MH160308
104	WLS17-JHK-104	Mahadebganj, Jharkhand	NEPZ	24.343	87.584	559211.90	2692359.80	22	1	MH160309
105	WLS17-JHK-105	Machut, Jharkhand	NEPZ	25.063	87.620	562485.70	2772018.10	23	1	MH160310
106	WLS17-HP-106	Lohar ghat, Himachal Pradesh	NHZ	31.190	76.838	675153.00	3452112.90	50	1	MH160311
107	WLS17-UK-107	Kashipur, Uttrakhand	NHZ	29.203	78.968	302468.50	3232131.60	51	1	MH160312
108	WLS17-UP-108	Ballia, Uttar Pradesh	NWPZ	28.200	79.361	339100.50	3120415.90	94	1	MH160313
109	WLS17-UP-109	Sultanpur, Uttar Pradesh	NWPZ	29.160	79.062	311507.30	3227320.80	95	1	MH160314
110	WLS17-UP-110	Mirzapur, Uttar Pradesh	NWPZ	25.133	82.551	656350.60	2780566.60	96	1	MH160315
111	WLS17-UP-111	Jagdishpur, Uttar Pradesh	NWPZ	26.749	80.542	454446.60	2958723.20	97	1	MH160316
112	WLS17-UP-112	Lucknow, Uttar Pradesh	NWPZ	26.766	80.977	497740.90	2960550.80	98	1	MH160317

*CZ, Central zone; NEPZ, North East Plain zone; NHZ, North hill zone; NWPZ, North Western Plain zone; HG, Haplotype group; HC, Haplotype Count*.

### Genomic Preparations

For genomic DNA extraction, isolates of UST were transferred, using a sterile needle, from Petri dishes to 100 ml Erlenmeyer flasks containing potato dextrose yeast (PDY) broth. The cultures were grown for 10 days in an orbital shaker (150 rpm) at 25 ± 1°C. The fungal mat harvested on sterile Whatman filter paper, frozen in liquid nitrogen, and ground to fine powder with pestle and mortar. Cetyl trimethyl-ammonium bromide (CTAB) method was used to extract genomic DNA as described by Kumar et al. (2013b). DNA was quantified by recording absorbance at 260 nm and determined purity by calculating the ratio of absorbance at 260 nm to that of 280 nm. The concentration of DNA was adjusted to 50 ng μl^−1^ for PCR analysis.

### RNA Polymerase II Second Largest Subunit (*RPB2*) Gene Amplification and Sequence Analysis

A portion of the RNA polymerase II second largest subunit (*RPB2*) gene was amplified for all the 112 UST isolates using polymerase chain reaction (PCR) with primer *RPB2*F (5′- AACCACCGATTTGGAGCAGT-3′) and *RPB2R* (5′- ACTCATTAGATGGCGGGGAGA-3′). The primers were designed using the NCBI accession number DQ846896.1. PCR amplification was performed in Q cycler 96 (Hain Lifescience, UK). Each PCR reaction mixture (50 μl) consisting of 50 ng template DNA, 1.5 μM of each primer, 1.5 mM MgCl_2_, 0.2 mM of each deoxynucleotides, 1.5 unit of *Taq* DNA polymerase (New England Biolabs, USA), and final volume of 50 μl was maintained by adding distilled water. The amplification was done as initial denaturing at 94°C for 4 min, followed by 35 cycles at 94°C for 1 min, annealing at 55°C for 1 min, extension at 72°C for 1 min and a final extension at 72°C for 7 min. The amplified PCR products were separated on 1% agarose gel, and the desired specific band was purified by Wizard SV Gel and PCR Clean-Up System (Promega, Madison, WI, USA) according to the manufacturer's instruction. DNA was sequenced commercially at the Eurofins Genomics India Pvt. Ltd. (Bangalore, India). A consensus sequence was obtained from the sequencing of both forward and reverse strands, and further data quality were checked using Chromas 2.32 (Technelysium Pty. Ltd.). BlastN search programme was used to compare the sequences available in National Center for Biotechnology Information (NCBI) databases. The sequences were aligned using MEGA 7 (Kumar et al., 2016) and gaps and missing data were not considered for phylogenetic analysis. Evolutionary tree was drawn using neighbor-joining (NJ) method (Saitou and Nei, 1987) and evolutionary distances were determined by Kimura (1980). The nucleotide sequences of *RPB2* gene were submitted to NCBI GenBank.

### Microsatellite Genotyping

For the amplification of each microsatellite marker gradient PCR was performed to select the best annealing temperature. PCR amplifications were performed in Q cycler 96 (Hain Lifescience, UK) in a total volume of 10 μl containing Promega™ PCR Master Mix, additional 0.5 mM MgCl_2_, 0.05–0.15 μM forward primer, 0.05–0.15 μM reverse primer, and 1.0 μl template DNA (50 ng μl^−1^). PCR cocktail without template DNA was taken as control. PCR was programmed for initial denaturation at 94°C for 4 min; followed by 35 cycles of denaturation at 94°C for 60 s, annealing at 51, 52, 53, 54, and 55°C for 1 min, and extension at 72°C for 1 min; with a final extension step at 72°C for 7 min. PCR products were separated in 4% agarose gel stained with ethidium bromide along 100 bp ladder (Genei, Bangalore) to know the polymorphism. Test isolates were scored on the basis of amplification and non-amplification of SSR markers. The numbers of varoius alleles per locus, effective alleles per locus, private alleles and Shannon's Information Index were computed for each population using GenAlEx 6.5 (Peakall and Smouse, 2012). The polymorphic information content (PIC) value for each SSR markers was calculated using the formula:

(1) $$\text{PIC}=1-\sum_{i=1}^{k}p_{i}^{2}$$

where k is the total number of alleles detected for a microsatellite; P_i_ is the frequency of the i^th^ allele in the set.

### Population Structure and Gene Flow

The presence (1) and absence (0) of desired amplicom for each SSR marker in all the 112 isolates were treated as binary characters and was analyzed using the NTSYS-pc program V2.1 (Rohlf, 2002). All the isolates were grouped in different clusters using Un-weighted Pair Group Method with Arithmetic average [UPGMA; (Yu et al., 2006)] in the SAHN subprogram. Dice similarity coefficient based on the proportion of shared alleles with the SIMQUAL was used to know the genetic similarity between isolates. Analysis of molecular variance (AMOVA) based on SSR markers was calculated using GenAlEx 6.5 to know the genetic diversity in different populations [Table 1; (Peakall and Smouse, 2012)]. The fixation index (F_st_) of the total populations and pairwise F_st_ among all pairs of populations were calculated to investigate population differentiation, and significance was tested based on 1,000 bootstraps. Gene flow among populations was calculated based on the number of migrants per generation (Nm) using the formula,

(2) $$\begin{array}{r} {\text{N}}_{\text{m}}=0.25\left(1-{\text{F}}_{\text{st}}\right)/{\text{F}}_{\text{st}} \end{array}$$

Population structure analysis was executed with STRUCTURE 2.3.4 (Pritchard et al., 2000) using microsatellite loci data. The optimum number of populations (K) was selected by testing *K* = 1 to *K* = 15 using five independent runs of 25,000 burn-in period length at fixed iterations of 100,000 with a model allowing for admixture and correlated allele frequencies. The optimum number of population was predicted using the simulation method of Evanno et al. (2005) in STRUCTURE HARVESTER version 0.6.92 (Earl and vonHoldt, 2012). The K value was determined by the log probability of data [Ln P(D)] based on the rate of change in LnP(D) between successive K. Bottleneck v1.2 was used to determine whether there was an excess (a recent population bottleneck) or deficit (a recent population expansion) in H (gene diversity) relative to the number of alleles present in UST populations (Piry et al., 1999). To determine whether loci displayed a significant excess or deficit in gene diversity Sign and Wilcoxon significance tests (WT) were performed (Cornuet and Luikart, 1996).

The Tajima's D value was estimated using the DnaSP program (Tajima, 1983). Number of haplotypes, number of segregating sites, and the π and Φw measures of nucleotide diversity for each population were determined with clone-corrected samples whereas, Nei's haplotype diversity (H_d_) was also determined before sample clone-corrected. The value of π and Φw represents the average number of pairwise nucleotide differences and the total number of segregating sites in a set of DNA sequences, respectively. MULTILOCUS version 1.31 was used to measure linkage disequilibrium among SSR loci using the index of association (I_A_) and rBar_D_ index (Agapow and Burt, 2001). Tests of departure from random mating for both indices were done with 10,000 randomizations of the complete and clone-corrected MLH dataset. To dissect the recombination in UST populations, the proportion of compatible pairs of loci (PrCP) was determined using MULTILOCUS v.1.31 (Agapow and Burt, 2001). The null hypothesis of random mating was rejected if more compatible loci than expected in a randomized population were observed (*P* < 0.05).

## Results

### Gene Sequence Analysis and Haplotypic Diversity

**Figure 1:**
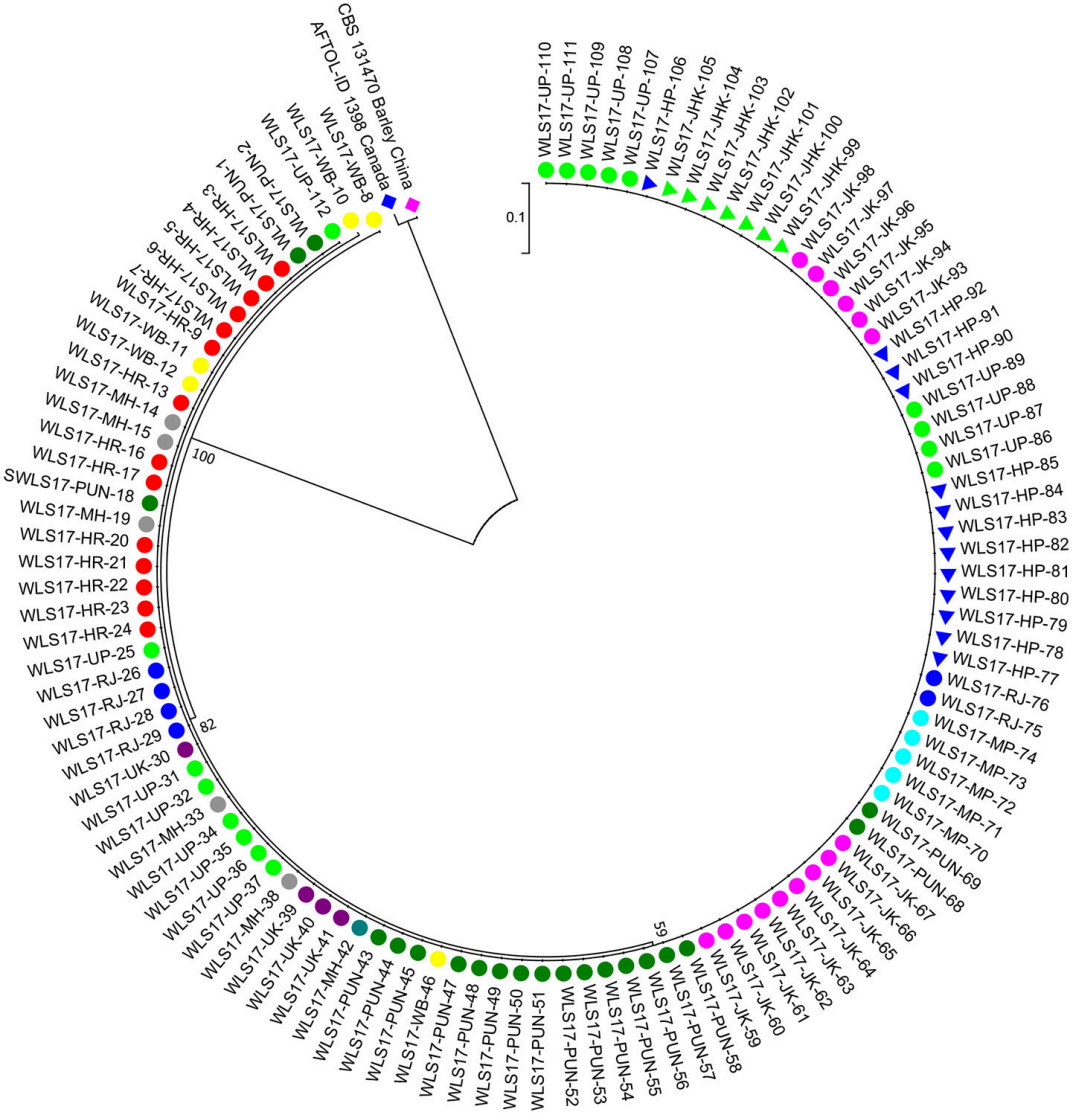
A neighbor-joining tree of RNA polymerase II gene (RPB2) sequences of the 112 isolates of UST from wheat growing states of India.

The second largest subunit of RNA polymerase II (*RPB2*) gene sequences of all the 112 UST isolates were compared with those from known species of *Ustilago* available in the NCBI database (Figure 1). The results confirmed that all the 112 isolates are *Ustilago segetum tritici*. Sequences were deposited at NCBI GenBank, and got accession numbers (Table 1). Phylogenetic analysis of *RPB2* gene from 112 isolates turn out from different wheat growing zones showed that they are related to each another and divergent from the isolate reported from Canada (Figure 1). Analysis of the sequences of the *RPB2* gene loci in the 112 isolates by DnaSP version 5.10 identified a total of 98 haplotypes (Table 1). Eighty three haplotypes were represented by single isolate and the remaining 15 haplotypes had minimum two isolates (Table 1). Fifteen haplotypes, which contained at least two isolates, were found only in NWPZ, CZ and NHZ. Haplotype 47 (WLS17-JK-93 and WLS17-JK-98) and haplotype 48 (WLS17-JK-94 and WLS17-JK-97) were collected from the NHZ while haplotype 53 (WLS17-HR-3, WLS17-HR-22 and WLS17-UP-88) and haplotype 55 (WLS17-HR-5, WLS17-UP-25 and WLS17-UP-34) were identified in NWPZ population only (Table 1). However, haplotype 24 (WLS17-UP-87 and WLS17-HR-20, WLS17-UK-30, WLS17-HP-78, and WLS17-JK-95) and haplotype 43 (WLS17-PUN-69 and WLS17-HP-85) were shared by NWPZ and NHZ populations. Similarly, CZ (WLS17-PUN-2) and NWPZ (WLS17-MH-14) shared haplotype 1, while haplotype 14 (WLS16-WB-11 and WLS17-HR-13) shared between NWPZ and NEPZ. NHZ and CZ shared haplotype 4 (WLS17-UK-41 and WLS17-MH-37). All the four populations displayed low nucleotide diversity (>0.0001) and haplotype diversity (>0.9905) (Table 2). CZ and NWPZ population possessed the highest haplotype diversity (1.000), while NEPZ population showed highest nucleotide diversity (0.00456) (Table 2). Five mutations were detected in entire populations (Table 2). NEPZ population showed highest number of singleton mutations. Further, estimates of DNA divergence between populations based on *RPB2* gene sequence analysis indicated that mutation events shared between UST population of CZ and NHZ and CZ and NWPZ (Table S1).

**Table 2:** DNA polymorphism data for *Ustilago segetum tritici* (UST) isolates based on *RPB2* gene sequence comparisons.

**DNA polymorphism parameter**	**Populations**				**Overall**
	**CZ**	**NEPZ**	**NHZ**	**NWPZ**	
Number of sequences	11	12	33	56	112
Selected region analyzed	1–687	1–687	1–687	1–687	1–687
Number of polymorphic sites (S)	2	4	2	3	5
Number of mutations (Eta)	2	4	3	3	5
Total number of singleton mutations, Eta(s)	1	4	1	3	4
Number of haplotypes (h)	11	12	29	51	98
Haplotype (gene) diversity (Hd)	1.0	1.0	0.9905	0.9955	0.9965
Nucleotide diversity (Pi)	0.00246	0.00456	0.00139	0.00053	0.00053
Theta (per sequence) from Eta	0.68283	1.32456	0.73919	0.65308	0.945
Theta (per site) from Eta	0.00330	0.00643	0.00357	0.00322	0.00471
Number of nucleotide differences (k)	0.50909	0.93939	0.28788	0.10714	0.107
Tajima's D	−0.77815	−1.02271	−1.39934	−1.68380	−1.85385
Fu and Li's D^*^ test statistic (FLD^*^)	−0.33034	−0.45895	−0.29252	−3.09074	−3.28229
Fu and Li's F^*^ test statistic (FLF^*^)	−0.45160	−0.68098	−0.71183	−3.10485	−3.20471
Fu's Fs statistic (FFs)	−0.659	0.560	−2.415	−4.521	−6.254
Minimum number of recombination events (Rm)	0	0	0	0	0

The population statistic parameters revealed statistically negative values of Tajima's D (−1.68380 to −0.77815) in UST populations of different wheat growing zones and provided evidence that the dominance of purifying selection and population expansion is operating in UST isolates (Table 2). Similarly, the test statistic FLD^*^ and FLF^*^ reflected analogous type of results for UST isolates and highlighted the principle of operation of purifying selection and population size expansion in different wheat growing zones. The statistically significant negative value of FFs statistic except NEPZ further strongly denotes the expansion observed in CZ, NWPZ, and NHZ population (Table 2).

### Marker Development, SSRs Polymorphism and Gene Diversity

To evaluate allelic diversity among UST isolates, 35 microsatellite markers were used. Twenty five SSR primer pairs produced clear single amplicons while rest did not amplify. Out of 25 primers, only 16 (Table 3) showed polymorphism and therefore used in genetic diversity analysis of 112 UST isolates originated from four geographical distinct zones of India. The polymorphism of the different SSR loci is presented in Table 3. The alleles per locus varied from two to four and allele size ranged from 170 to 750 bp. The PIC values ranged from 0.3713 to 0.6632. One SSR loci (UST31) was highly informative (PIC ≥ 0.5) and rest all loci were reasonably informative (0.5 < PIC > 0.25). The 16 polymorphic primer pairs revealed a total of 68 alleles across the 34 loci in 112 isolates, ranging from 2 to 4 alleles per isolate (Table 3). The markers were all selectively neutral according to the Ewens-Watterson test (Table S2).

**Table 3:** Characteristics of sixteen neutral microsatellite markers used in the study for population genetic diversity analysis of *Ustilago segetum tritici* (UST) isolates.

**S. No**.	**Sequence (5^**′**^-3^**′**^)**	**Motif**	**Amplicon size (bp)**	**Number of allele**	**Ta (^**°**^C)**	**He**	**PIC**
1	USTF 5: GCTCGTCTACCTCTGCGATACT	(GGA)_5_	215–241	2	55	0.4984	0.3742
	USTR 5: TCTGCATCTCAATCAACCAATC						
2	USTF 6: AGACATGCACCGTAACAACAAC	(CGG)_6_	180–200	2	55	0.4986	0.3743
	USTR 6: TACCCTCCATACTCTTGTCCGT						
3	USTF 7: AAGCATACTCAAGGCAGGGTAA	(CAT)_5_	170–240	2	54	0.4854	0.3676
	USTR 7: GTTCTCGGATGGTCTCGTCTAC						
4	USTF 14: AAAAGTCATCCTCGTTTCGGTA	(CCG)_6_	180–230	2	55	0.4926	0.3713
	USTR 14: AGATAGGGAAGCAAATCATGGA						
5	USTF 16: GCCTCTTCATCTCTCTCCTCAC	(GAC)_6_	220–270	2	52	0.4948	0.3724
	USTR 16: TGACTCTTCTGCATCATATCGG						
6	USTF 17: TCTTGTGGAGTCTGCTGTTGTT	(TGC)_5_	230–250	2	52	0.4991	0.3746
	USTR 17: GTAGCTTCAGGTCGCATCACTT						
7	USTF 23: TCGTGAAAACTAACAGAGCCAA	(AAAT)_4_	220–250	2	52	0.498	0.374
	USTR 23: ACACCTATTTGCGTGAAGGAGT						
8	USTF 25: TACTTCTCCTCCTCCTCCTCCT	(TATT)_5_	285–300	2	52	0.4997	0.3748
	USTR 25: GAACTCGCAAAGTGGTTTCTCT						
9	UST 26: AGAGACCAAGTCGAATCCAAAG	(CAAGG)_6_	294–320	2	52	0.4991	0.3746
	USTR 26: CCTTGCCTACTTCTCCCTACCT						
10	USTF 27: CATTTCAGTGTTGGACAAGCAT	(GTGTCA)_4_	250–300	2	54	0.4959	0.3729
	USTR 27: AGAGAGTTTCGTAGTTGGGCAG						
11	USTF 30: GGTGATTGGAAGACCACAGAAT	(AAGCCA)_5_	227–250	2	55	0.4988	0.3744
	USTR 30: GTTTTGAACTCTCTGCTTTGGG						
12	USTF 31: CACAAACACACACACACACACA	(GCTCCC)_4_	224–750	4	54	0.717	0.6632
	USTR 31: CTGAACAGTAAAGCCTGAAGGG						
13	USTF 32: TCCTACATTGGGATGACTGATG	(CAACGG)_4_	217–250	2	52	0.4975	0.3737
	USTR 32: GACTCGCTTCTTGTTCTTGGTT						
14	USTF 33: GAAAGAGAGAGGGAGGGAAGAG	(GGAGAA)4	230–250	2	53	0.4991	0.3746
	USTR 33: TGCGTATAGGTATGTGTGGCTT						
15	USTF 34: GAAGAAAATGCTAGAGCGAAGG	(CA)8	216–250	2	55	0.4973	0.3737
	USTR 34: AGCAGAAGGTGAGAGAGCGTAT						
16	USTF 36: ATGAGGTCAAGAGTCAGCAACA	(GA)8	175–200	2	54	0.4978	0.3739
	USTR 36: ATTCGTCAAGATGCCTTTCACT						

### Population Genetic Diversity

The genetic diversity indices for diverse four UST populations from different wheat growing zones are depicted in Table 4. The number of different alleles (N_a_), effective alleles (N_e_) and expected heterozygosity (H_e_) averaged across all loci ranged from 1.824 to 2.0, 1.695 to 1.721, and 0.373 to 0.406, respectively for the four different populations (CZ, NEPZ, NHZ, and NWPZ). The NWPZ population had the highest N_a_ values (2.000) while the CZ population had the lowest (1.765). Expected heterogyzosity (H_e_) and effective Alleles (N_e_) values were lowest in CZ population (H_e_ = 0.373; N_e_ = 1.695) and the highest in NWPZ population (H_e_ = 0.406; N_e_ = 1.729). Similarly, unbiased gene diversity (uH_e_) was the lowest in NHZ population (uH_e_ = 0.381) and the highest in NWPZ population (uH_e_ = 0.410), with an average of 0.396. The polymorphic loci (%) fell in the range of 88.24% (NHZ and NEPZ) to 100% (NWPZ), with an average of 88.35% (Table 4). However, NHZ and NWPZ population showed the highest number (17) of polymorphic loci. Information index based on the Shannon's Index (I), the highest diversity was recorded in NWPZ (*I* = 0.589) population, whereas it was lowest in CZ (*I* = 0.531) population. Of all the analyzed populations, no private allele was found in entire population. The genetic diversity within populations (H_s_) ranged from 0.0501 to 0.4943 with an average of 0.3367 (Table S2), and responsible for 91% of the total genetic diversity (H_T_ = 0.3611). The proportion of the total genetic diversity attributable to the population differentiation (G_st_) ranged from 0.0029 to 0.391 with an average of 0.0678 over all loci (Table S2).

**Table 4:** Summary of the population diversity indices calculated on the basis of SSR markers.

**Zone**	***N***	**N_**a**_**	**N_**e**_**	**I**	**H_**e**_**	**uHe**	**PL (%)**	**Number of polymorphic bands**	**Least common bands (≤5%)**
CZ	11	1.765 ± 0.136^*^	1.695 ± 0.088	0.531 ± 0.063	0.373 ± 0.045	0.391 ± 0.047	82.35	16	16
NEPZ	12	1.824 ± 0.128	1.721 ± 0.085	0.552 ± 0.056	0.387 ± 0.041	0.403 ± 0.043	88.24	16	16
NHZ	33	1.882 ± 0.081	1.697 ± 0.086	0.536 ± 0.060	0.375 ± 0.044	0.381 ± 0.044	88.24	17	16
NWPZ	56	2.000 ± 0.000	1.729 ± 0.064	0.589 ± 0.032	0.406 ± 0.027	0.410 ± 0.027	100.00	17	17
Mean		1.868 ± 0.051	1.710 ± 0.040	0.552 ± 0.027	0.385 ± 0.020	0.396 ± 0.020	82.35		

*N, number of isolates; N_a_, No. of different Alleles; N_e_, No. of Effective Alleles = 1/(p^2^ + q_2_); I, Shannon's Information Index; He, Expected Heterozygosity = 2 X pX q; uH_e_, Unbiased Expected heterozygosity = [2N/(2N-1)] X H_e_; PL (%) = Percent Polymorphic Loci*.

* *The values after ± denote the standard errors*.

*CZ, Central zone; NEPZ, North East Plain zone; NHZ, North Hill zone; NWPZ, North Western Plain zone*.

### Population Genetic Structure and Gene Flow

The AMOVA analysis comparing the four populations showed that 1% of the total variance was distributed among zones. A relatively higher proportion of the variation (91 %) was distributed within UST isolates (Table 5). Genetic variation among wheat growing zones (F_st_ = 0.012), isolates (F_is_ = 0.079) and within isolates (F_it_ = 0.090) was mentioned in Table 5. Pairwise F_st_ values of the genetic distance between different populations were low but significant (*P* < 0.01; Table 6).

**Table 5:** Summary of the analysis of molecular variance (AMOVA) results for 112 isolates of *Ustilago segetum tritici* (UST) (*n* = 112).

**Source**	**df**	**SS**	**MS**	**Estimated variance**	**Percentage %**	**F-Statistics**	**Value**	**P**
Among population	3	16.457	5.486	0.039	1%	Fst	0.012	0.606
Among individuals	108	388.048	3.593	0.263	8%	Fis	0.079	0.019
Within individuals	112	343.500	3.067	3.067	91%	Fit	0.090	0.606
Total	223	748.004		3.369	100%			

*Df, degree of freedom; SS, sum of squared observations; MS, mean of squared observations; F_st_ = AP/(WI + AI + AP) = AP/TOT; Fis, AI/(WI + AI); Fit, (AI + AP)/(WI + AI + AP) = (AI + AP)/TOT; Nm = [(1/Fst)-1]/4] = 21.0. Key: AP , Est. Var. Among zones, AI Estimated variance among isolates, WI = Estimated variance within isolates*.

**Table 6:** Measure of the pair-wise comparisons of genetic distance (F_st_), genetic flow (Nm) and Nei's unbiased genetic identity for *Ustilago segetum tritici* (UST) isolates collected from the four zones of India.

**Population 1**	**Population 2**	**F**_****st****_		**N_**m**_**	**Nei's unbiased genetic identity**
		**SSR loci**	***RPB2* gene**		
NEPZ	CZ	0.016	0.045	14.989	0.9358
NEPZ	NWPZ	0.009	0.041	4.934	0.944
NEPZ	NHZ	0.037	0.045	6.499	0.9514
CZ	NWPZ	0.000	0.000	0.00	0.9907
CZ	NHZ	0.048	0.000	29.131	0.9781
NHZ	NWPZ	0.000	0.000	0.00	0.9947

*CZ, Central zone; NEPZ, North East Plain zone; NHZ, North hill zone; NWPZ, North Western Plain zone*.

The average gene flow among populations (N_m_) was ranged from 0.00 (between NWPZ and NHZ; between NWPZ and CZ) to 29.131 (between CZ and NHZ). Pairwise estimates of gene flow (N_m_) indicated that Nm value was more than 1.0 in most of the population pairs suggesting gene flow between populations, although with different magnitude except NWPZ and NHZ and NWPZ and CZ (Table 6). The highest value was observed among pair of CZ and NHZ populations (N_m_ = 29.131; F_st_ = 0.048) followed by CZ and NEPZ pair (N_m_ = 14.989; F_st_ = 0.016). When population of NEPZ was compared with other populations for genetic distance (F_st_) on the basis of SSR (0.009–0.037) and *RPB2* gene loci (0.041–0.045) and gene flow (N_m_ = 4.934–14.989) indicating these populations are differentiated with low gene flow. Pair wise comparison of NHZ and NWPZ recorded the highest indices of Nei's unbiased genetic identity (0.9947), while CZ and NHZ showed lowest levels of genetic identity (0.9358) (Table 6).

**Figure 2:**
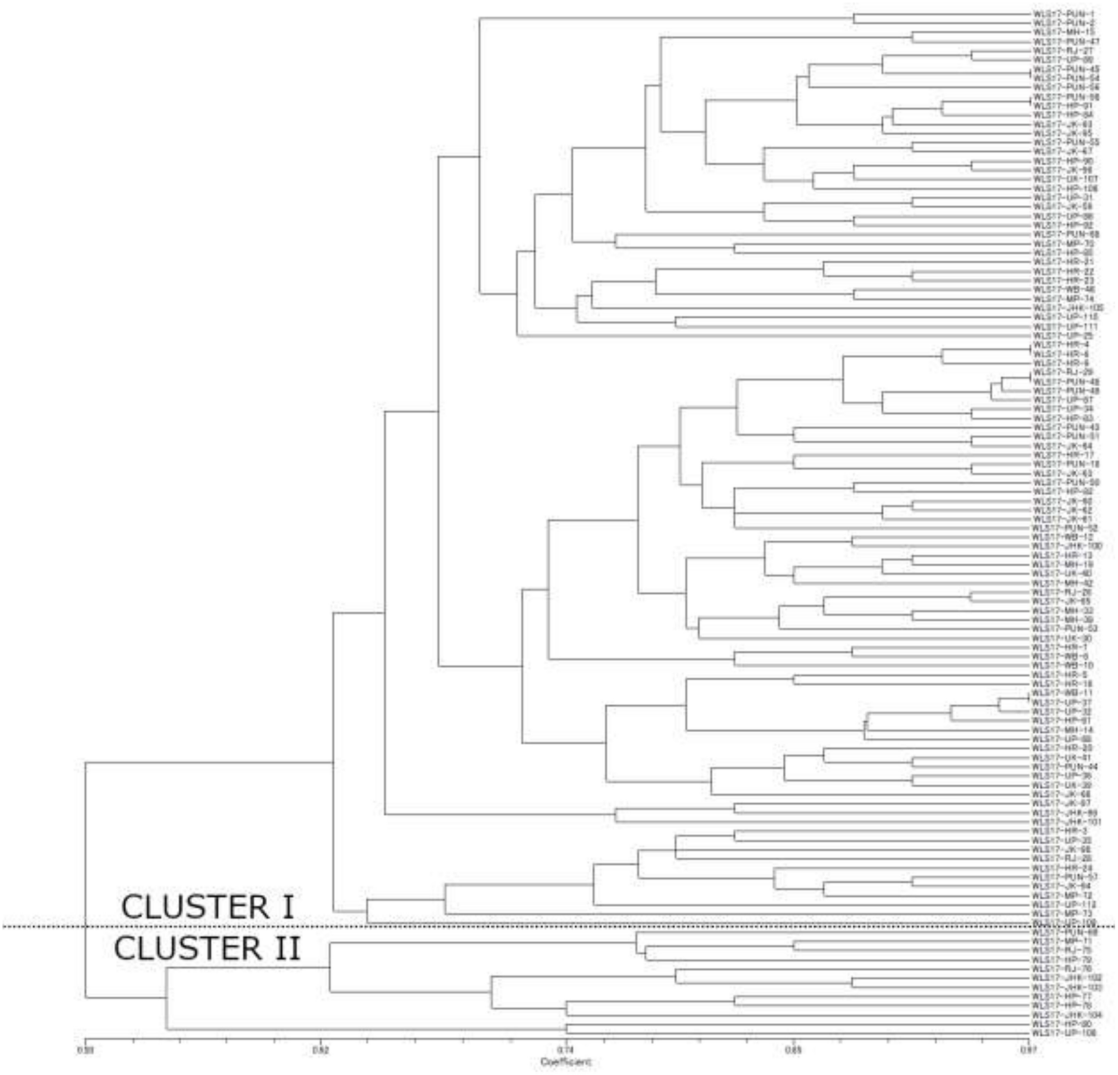
Unweighted Neighbor-joining tree using the simple matching similarity coefficient based on 16 microsatellite markers for the 112 isolates of UST isolated from wheat spikes in India.

**Figure 3:**
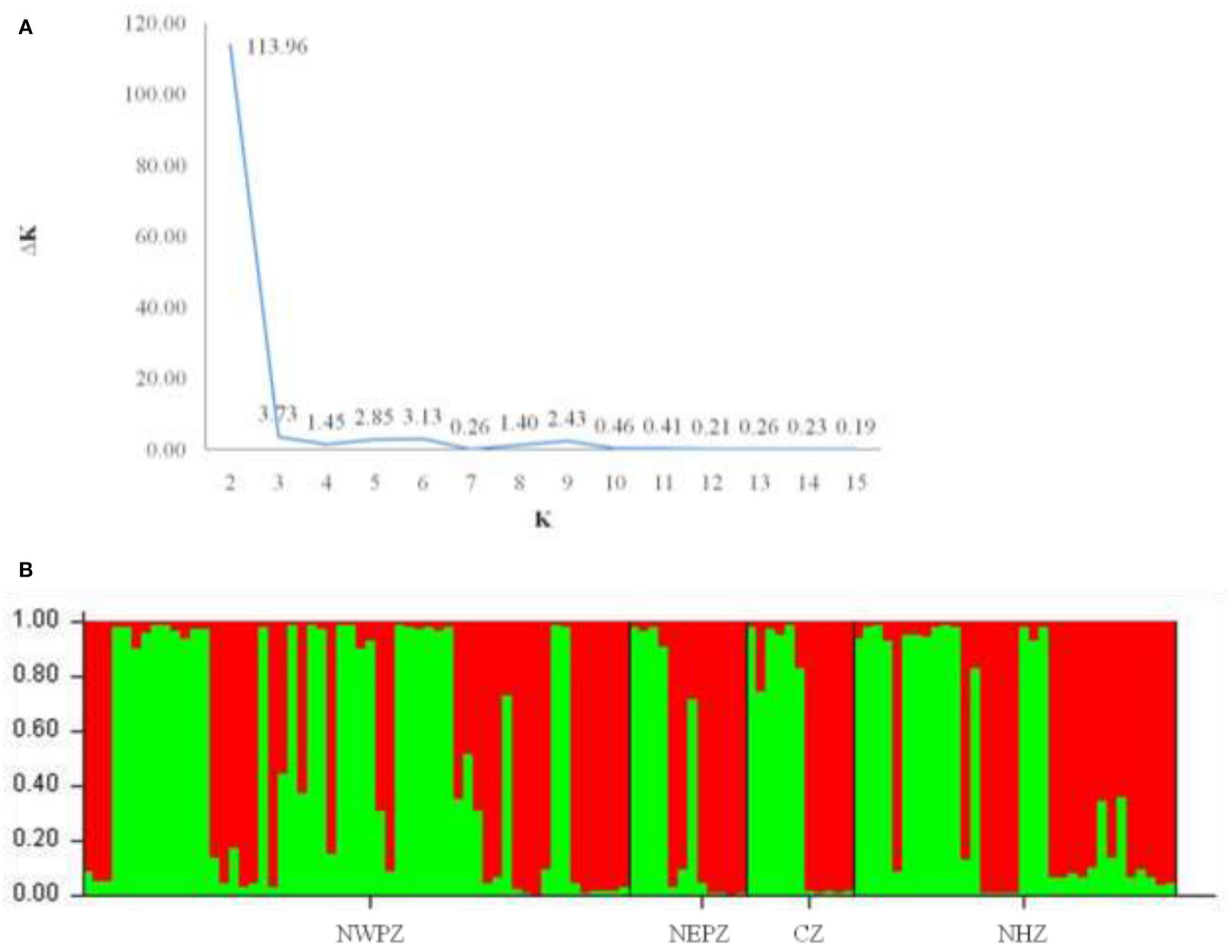
Cluster analyses of UST populations from four different wheat growing zones of India (results from Structure v2.2). **(A)** Each isolate is represented by a bar, divided into K colors, where *K* = 2 is the number of clusters assumed. Individuals are sorted according to Q, the inferred clusters; **(B)** Magnitude of ΔK calculated for each level of K. Maximum ΔK indicates the most likely number of UST populations (*K* = 2).

The dendrogram based on unweighted Neighbor-joining method grouped all the 112 isolates representing four populations into two major clusters (Figure 2). Among these, 100 and 12 isolates were grouped in cluster 1 and cluster 2. The grouping by UPGMA using genetic distances do not showed any spatial clustering among the different geographic zones (Figure 2). Several subgroups within cluster 1 were observed irrespective to populations, indicating genetic variability within and among isolates in each population. The similarity coefficient of overall isolates averaged 0.50. The substructure analysis for genetic relationship among UST isolates, excluding loci with null alleles, showed a clear ΔK peak at *K* = 2 (ΔK = 113.96) (Figure 3A) and *K* = 2 was the most likely value thus revealed that all individuals grouped into two major clusters (Figure 3B).

### Linkage Disequilibrium and Population Expansion

Linkage disequilibrium analysis was performed to infer the reproductive strategy. The values of I_A_ (0.991–2.034) and rbar_D_ indices (0.066–0.134) in the association tests differed significantly from zero in all the UST populations (Table 7). UST isolates sampled from different wheat growing zones rejected the null hypothesis of gametic equilibrium, this shows that isolates in each zone were not under random mating (Table 7).

**Table 7:** Estimation of linkage disequilibrium by the index of association (I_A_) and the unbiased (rd) statistic of Indian *Ustilago segetum tritici* (UST) isolates.

**Population**	**I_**A**_**	**rBar_**D**_**	***P*-Value**
CZ	1.399	0.092	< 0.001
NEPZ	2.034	0.134	< 0.001
NHZ	0.991	0.066	< 0.001
NWPZ	1.281	0.082	< 0.001
Total	1.169	0.075	< 0.001

Index of association (I_A_) and rBar_D_, a modified index of association (1); P-values were estimated from 1,000 randomizations and are identical for I_A_ and rBar_D._

The results depicted in Table 8 shows that all the UST populations evolved through stepwise mutation method (SMM). The sign tests in Bottleneck revealed a significant H deficit in 1 of the 13 loci under IAM (*P* = 0.00027), TPM (*P* = 0.00070) and SMM (*P* = 0.00132) model of evolution, indicating recent population expansion in CZ. Similarly, in NHZ and NWPZ population, significant H deficit in 1 of the 14 loci under IAM, TPM, and SMM model of evolution were observed (Table 8).

**Table 8:** Comparison of observed genotypic diversity (H) and expected genotypic diversity (HEQ) at mutation-drift equilibrium based on the observed number of alleles with infinite number of alleles.

**Zone**	**Test**	**Parameter**	**IAM**	**TPM**	**SMM**	**Mode**
CZ	ST	Number of loci with heterozygosity excess expected	6.35	6.86	7.22	Shifted
		Loci with heterozygosity deficiency or excess	1/13 (*P* = 0.00027)	1/13 (*P* = 0.00070)	1/13 (*P* = 0.00132)	
	SDT	T2 values (Probability)	4.024 (*P* = 0.00003)	3.492 (*P* = 0.00024)	3.168 (*P* = 0.00077)	
	WT	One tail for H deficiency	*P* = 0.99997	*P* = 0.99994	*P* = 0.99991	
		One tail for H excess:	*P* = 0.00006	*P* = 0.00009	*P* = 0.00015	
		Two tails for H excess and deficiency:	*P* = 0.00012	*P* = 0.00018	*P* = 0.00031	
NEPZ	ST	Number of loci with heterozygosity excess expected	6.53	6.82	7.22	Shifted
		Loci with heterozygosity deficiency or excess	0/14 (*P* = 0.00002)	1/13 (*P* = 0.00064)	1/13 (*P* = 0.00130)	
	SDT	T2 values (Probability)	5.195 (*P* = 0.00000)	4.575 (*P* = 0.00000)	4.182 (*P* = 0.00001)	
	WT	One tail for H deficiency	*P* = 1.00000	*P* = 0.99997	*P* = 0.99997	
		One tail for H excess:	*P* = 0.00003	*P* = 0.00006	*P* = 0.00006	
		Two tails for H excess and deficiency	*P* = 0.00006	*P* = 0.00012	*P* = 0.00012	
NHZ	ST	Number of loci with heterozygosity excess expected	6.32	6.75	7.10	Shifted
		Loci with heterozygosity deficiency or excess	1/14 (*P* = 0.00005)	1/14 (*P* = 0.00012)	1/14(*P* = 0.00024)	
	SDT	T2 values (Probability)	5.158 (*P* = 0.00000)	4.635 (*P* = 0.00000)	4.197 (*P* = 0.00001)	
	WT	One tail for H deficiency	*P* = 0.99997	*P* = 0.99995	*P* = 0.99962	
		One tail for H excess:	*P* = 0.00005	*P* = 0.00008	*P* = 0.00050	
		Two tails for H excess and deficiency	*P* = 0.00009	*P* = 0.00015	*P* = 0.00101	
NWPZ	ST	Number of loci with heterozygosity excess expected	6.01	6.44	6.81	Shifted
		Loci with heterozygosity deficiency or excess	1/14(*P* = 0.00003)	1/14 (*P* = 0.00006)	1/14 (*P* = 0.00014)	
	SDT	T2 values (probability)	5.703 (*P* = 0.00000)	5.161 (*P* = 0.00000)	4.728 (*P* = 0.00000)	
	WT	One tail for H deficiency	*P* = 0.99998	*P* = 0.99998	*P* = 0.99998	
		One tail for H excess:	*P* = 0.00003	*P* = 0.00003	*P* = 0.00003	
		Two tails for H excess and deficiency	*P* = 0.00006	*P* = 0.00006	*P =* 0.00006	

*CZ, Central zone; NEPZ, North East Plain zone; NHZ, North Hill zone; NWPZ, North Western Plain zone; ST, Sign rank Test; SDT, Standardized Differences Test; WT, Wilcoxon Test; IAM, Infinite allele model; TPM, Two Phase Model; SMM, Stepwise Mutation Model of Evolution*.

*Probability of deviation from mutation-drift equilibrium (H>HEQ) was determined according to Cornuet and Luikart (1996)*.

The bottleneck analysis supported for the non-existence of any bottleneck in UST populations in recent past. The concept of heterozygosity excess works on the principle that the observed gene diversity is higher than the expected equilibrium gene diversity (Heq) in a recently bottlenecked population (Table 8). In CZ population, sign rank test under IAM mutation model, expected number of loci with heterozygotic excess was 6.35 while the observed number of loci with heterozygosity excess was 13 (Table 8). The expected and observed loci with heterozygosity excess calculated by using TPM and SMM models were 6.86 and 7.22, respectively. Similarly, the outcome for IAM, TPM, and SMM supported the absence of any bottleneck in CZ population. Similar trends were also observed for NHZ, NEPZ, and NWPZ population using TPM and SMM. Although, one locus with heterozygosity deficiency (*P* = 0.00002) was also observed in NEPZ population using IAM model. The SDT provided the T2 statistics equal to 4.024, 3.492, and 3.168 for IAM, TPM, and SMM, respectively in CZ population. The probability values were significant for IAM (*P* = 0.00003), SMM (*p* = 0.00024), and TPM (*P* = 0.00077). Thus, null hypothesis was accepted by all the models. The probability values with WT for one tail for H excess under three models IAM (*p* = 0.00006), TPM (*p* = 0.00009), and SMM (*p* = 0.00015) indicated acceptance of null hypothesis under all the models in CZ population. Thus, all the three tests (ST, SDT, and WT) indicated the acceptance of mutation drift equilibrium (*P* > 0.05) in UST populations under all the mutation models for all CZ, NHZ, NEPZ, and NWPZ populations. Another powerful test of qualitative graphical method based on the allele frequency spectra detected a normal L-shaped curve, where the alleles with the lowest frequencies (0.03–0.3) were found to be most abundant in the entire wheat growing zones (Figure S2).

## Discussion

Loose smut is a monocyclic internally seed borne disease. The seed borne inocula leads to long distance rapid spread of the disease across the entire wheat growing zones of India. The *Ustilago segetum tritici* isolates were collected from four major wheat growing zones (NWPZ, NEPZ, CZ, and NHZ) of India. The genetic structure was analyzed by performing *RPB2* gene sequence comparison and fingerprinting with newly developed SSR markers. The degree of nucleotide difference in the *RPB2* region in UST populations is low. It may be due to action of concerted evolution leads to homogenizing effect. Furthermore, evidence of recombination (R_m_ = 0) in entire UST population was not detected.

The low but significant F_st_ values (< 0.01 and < 0.05) and pair wise population differentiation among UST population from different zones indicate low genetic differentiation in the total populations (Fst = 0.012). The UST populations appear either of common origin or limited distribution, reproduces predominantly by asexual means, or experience substantial gene flow (from CZ to NHZ and NEPZ and later from NEPZ to NWPZ and NHZ) coupled with genetic drift. However in populations where mutation rates are high, F_st_ tends to fall back to zero (in case of CZ) as novel alleles are added to the population (Onaga et al., 2015). This happened because of negative dependence of F_st_ on diversity (Charlesworth et al., 1997) and has been reported in several pathogens; even in the absence of asexual reproduction (Couch et al., 2005). To outwit this prejudice due to mutation rates, F_ST_ has been compared with other genetic diversity indices. Analysis of molecular variance (AMOVA) showed that 91% of the total variation was due to differences among isolates within populations and the variation among populations reflected only 1% of the total variation. A low degree of differentiation among UST populations may be due to admixture among isolates from the different geographic regions. These results are also unswerving with the low Shannon's indices (0.531–0.589). The overall Shannon's index (*I* = 0.552) suggests that more than 50% of the genetic diversity explained by the differences between isolates. Therefore, all these results conclude that most of the genetic variation (91%) was distributed among isolates across the regions. The similar findings have been earlier reports in *Ustilago maydis* (Valverde et al., 2000), *Mycosphaerella graminicola* (Boeger et al., 1993), *Phytophthora infestans* (Goodwin et al., 1994), *Rhynchosporium secalis* (McDonald et al., 1999), and *Rhizoctonia solani* (Goswami et al., 2017) while analyzing their populations structure. It is worth mention here that the fungal isolates are mostly similar at the genetic level despite long distances among different wheat growing zones in India.

Haplotype analysis performed in present study provides information on the number of haplotypes (h), their frequency and diversity, and genetic distances within and between *RPB2* gene sequence. The H_d_ can range from zero to 1.0, which means no diversity to high levels of haplotype diversity (Nei and Tajima, 1981). In present study, H_d_ (0.104–0.473) values indicated low to moderate levels of diversity in different wheat growing zones. NWPZ revealed the maximum diversity based on the number of haplotypes (i.e., 51 haplotypes from 56 UST isolates). Few haplotypes were shared among different populations indicating role of asexual reproduction and long-distance dispersal. Contrary to this, sexual reproduction occurs at the site of infection on the spike, was apparently prevalent in Indian wheat growing areas. Besides this, two other reasons may explain the genetic variability among isolates, first there might be possibility of multiple founder populations that result in the admixture of populations. Secondly, population genetic expansion may took place due to the accumulation of different alleles in UST populations as evident from the total and shared mutations noticed in the presents study. All these are agreeable, as evidenced by the high levels of population admixture identified in Structure analysis. Mutation generates diverse regional populations of UST, creating a pool of mutants from which new, virulent isolates can emerge. Many haplotypes were shared among these populations supports multiple introductions of the same haplotype, which could be due to pronounced asexual reproductive phase, as the case with UST. Furthermore, the analysis revealed that all UST populations are admixed and contain haplotypes from multiple populations within a region or between the regions.

To investigate the role of evolutionary forces on UST population, different neutrality test statistics (Tajima's D, Fu and Li's D^*^ and F^*^, and Fu's Fs) were performed to examine the *RPB2* sequence data for departure from neutrality. Significant and negative Tajima's D test statistics indicated that *RPB2* locus is experiencing population bottlenecks, where the population is largely uniform and only a few sequences compose the new population. The biology of UST and its colonization could serve as a source of population bottlenecks. Similarly, significant and negative value of almost all D^*^ and F^*^ test statistics showed strong purifying selection. Overall, the *RPB2* gene data displayed genetic divergence in the structure of the population among the four wheat growing zones analyzed and well-supported by the results from microsatellite loci. Moreover, 16 newly developed SSR markers used were polymorphic on all of the UST isolates. These results are comparable to earlier reports on SSR markers developed for other plant pathogens (Yang and Zhong, 2008; Pouzeshimiab et al., 2014). Thus, these SSR markers could be useful tool to study the population biology and genetics of this fungus at global level.

The loose smut fungus is carried as dormant mycelium within healthy appearing seed and is spread by growing infected seed. Moreover, teliospores are easily shaken from the smutted heads and may be carried for long distances by wind, insects, or other agencies (Ram and Singh, 2004). The low differentiation among regions (8% of total variation) detected on the basis of microsatellites can be explained by different ways. Firstly, the level of gene flow (N_m_) is sufficient to maintain genetic similarity. The low levels of population differentiation were observed in corresponding high values of N_m_. N_m_ is >1, reveals little differentiation among populations, and under such circumstances migration is more important than genetic drift (McDermott and McDonald, 1993). Theoretically, average gene flow value (N_m_ = 21) indicates that 21 isolates would need to be exchanged each generation among populations of different regions to achieve current degree of similarity. Highest gene flow was recorded between the CZ and NHZ (N_m_ = 29.131) populations. Regular gene flow and random mating among isolates from various populations could result in new pathotypes with improved pathological and biological fitness traits (Mishra et al., 2006). Besides this, inbreeding coefficient (F_it_ = 0.090) indicated little genetic differentiation across UST populations. The corroborating results were also observed in American populations of *P. nodorum* (F_st_ = 0.004; Stukenbrock et al., 2006), North American population of *Mycosphaerella graminicola* (F_st_ = 0.08) (Zhan et al., 2003) and *Septoria musiva* populations (F_st_ = 0.20) in north-central and northeastern North America (Feau et al., 2005). In UST populations, SSR data provided no discrete clustering in different populations on the basis of structure analysis and little among-populations variance (8%) was observed in AMOVA. Therefore, no specific demarcation of genetic grouping was noticed, and results further suggest large and widespread populations with high migration rates facilitated by wind-dispersed teliospores and frequent exchange and long distance transport of infected seed material in different wheat growing zones.

In present study, the plausible reasons leading to the structuration of the regional collection were not elucidated since no clear main direction of gene flow among the sampled sites, and no significant isolation by distance (*P* = 0.49; > 0.05) were observed. The genetic identities of the four populations evaluated in present study were close to 1. The moderate G_st_ (−0.0678) values indicated weak genetic differentiation and minimal geographic clustering among the populations from four different zones and yielded average Nm values 6.875 across all loci and populations, suggesting that the level of gene flow was approximately seven times greater than that needed to prevent populations from diverging by genetic drift. Moreover, absence of private alleles in all the four zone population may indicate that the observed migration levels reflect gene flow. The wind direction in India is generally from North West to North East during the wheat cropping season. This may cause migration and gene flow between populations lead to admixture among isolates from the diverse geographic origin as observed in present study. The pathogen is cosmopolitan in distribution, and telisopores are known to be disseminated over long distances by wind (Ram and Singh, 2004). Besides this, long-distance gene flow in UST was man mediated, and subsequent natural gene flow gradually reduced the isolation by distance.

Mutation is the main evolutionary mechanism that generates polymorphisms, and its implications to disease management are well-known (Jolley et al., 2005). For UST, point mutations (4 mutations) in the sequence of the *RPB2* gene resulted in the introduced of new alleles in the population. Similar observations have been documented by Lourenço et al. (2009), while studying the molecular diversity and evolutionary processes of *Alternaria solani*, a seed borne pathogen in Brazil inferred using genealogical and coalescent approaches. However, the relatively low number of singleton mutation estimated in present study for *RPB2* locus in different wheat growing zones does not signify low mutation rate in whole genome. Therefore, authors felt that the evaluation of other housekeeping genes or genomic regions should be analyzed for more accurate quantification of mutation occurrence in UST populations. Further, bottleneck analysis based on three models (IAM, TPM and SMM) indicated that the observed heterozygosity excess (H_e_) found less than the expected excess heterozygosity (H_ee_) in all the four wheat growing zones. Thus, the lower magnitude of H_e_ with their respective H_ee_ reflect absence of genetic bottleneck in UST populations. Further, the negative TD-value of the UST population indicates that the UST population is undergoing demographic expansion. Further support for this hypothesis is gained from lack of private alleles in UST populations collected from all wheat growing zones.

The knowledge of population genetic structure of a pathogen provides information on its potential to overcome host genetic resistance (McDonald and Linde, 2002). The results of present study showed that UST have a clonal genetic structure with limited differentiation between populations. It means variability is mainly contributed by mutation and recombination is uncommon. Therefore, wheat disease management measures, such as replacement of infected seed and fungicide-treated seeds, could help to reduce UST severity and limit gene flow. Host resistance is also economical and effective to manage loose smut of wheat (Singh, 2018), and breeding efforts in different wheat growing zones have put emphasis on exploration of more resistance sources and other gene pools to fill this gap. In addition, screening of germplasm and breeding material against genetically diverse isolates needs to be emphasized to develop durable and effective resistance cultivars. In nutshell, the current study presents a first stab to comprehend the genetic variation within and among populations of UST causing loose smut in different wheat growing regions. The results highlight that microsatellite markers can be used to analyze genotypic and genetic diversity of populations of UST.

## Author Contributions

The work was conceived and designed by PK and SK. The sampling survey was performed by PK, SK, PJ, and DS. Experiments were conducted by RT, PK, and RK. Data analysis was done by PJ and RT. The manuscript was drafted by PK and SK. The final editing and proofing of manuscript was done by DS and GS. RK and RT contributed equally. The manuscript was approved by all the authors.

### Conflict of Interest Statement

The authors declare that the research was conducted in the absence of any commercial or financial relationships that could be construed as a potential conflict of interest.
