# Supplementary material for: Phylogeography and Population Structure Analysis Reveal Diversity by Gene Flow and Mutation in Ustilago segetum (Pers.) Roussel tritici Causing Loose Smut of Wheat
Source: Front Microbiol. 2019 May 15;10:1072. doi: 10.3389/fmicb.2019.01072 (PMC6529584; doi:10.3389/fmicb.2019.01072)
Supplement: Supplementary file 1 [file Data_Sheet_1.docx]

| **Table S1:** Estimates of DNA Divergence between populations based on *RPB2* gene sequence analysis | | | | | | | | |
| --- | --- | --- | --- | --- | --- | --- | --- | --- |
| Population 1 | Population 2 | Shared mutation | Total Number of mutation | ANd | Dxy | Da | k | Pi(t) |
| CZ | NEPZ | 0 | 6 | 0.758 | 0.00368 | 0.00016 | 0.751 | 0.00365 |
| CZ | NHZ | 1 | 1 | 0.116 | 0.00056 | -0.00003 | 0.089 | 0.00043 |
| CZ | NWPZ | 1 | 3 | 0.229 | 0.00113 | 0.00006 | 0.147 | 0.00072 |
| NEPZ | NHZ | 0 | 5 | 0.525 | 0.00256 | 0.00012 | 0.305 | 0.00149 |
| NEPZ | NWPZ | 0 | 6 | 0.387 | 0.00191 | 0.00007 | 0.205 | 0.00101 |
| NHZ | NWPZ | 0 | 2 | 0.036 | 0.00018 | 0.00000 | 0.045 | .00022 |
| Dxy: Average number of nucleotide substituted per site between populations; Da: Number of net nucleotide substituted per site between populations, ANd: Average number of nucleotide differences between populations; k= Average number of nucleotide differences; Pi(t): Nucleotide diversity | | | | | | | | |

| **Table S2: Diversity indices of the microsatellite loci used in the study** | | | | | | | | |  |  |  |  |  |
| --- | --- | --- | --- | --- | --- | --- | --- | --- | --- | --- | --- | --- | --- |
| **Locus** | **n_a_** | **ne** | **H** | **I** | **H_t_** | **H_s_** | **G_st_** | **N_m_** | **Ewens-Watterson Test for Neutrality** | | | | |
|  |  |  |  |  |  |  |  |  | **Obs. F** | **Min F** | **Max F** | **L95^a^** | **U95^a^** |
| UST1 | 2 | 1.0364 | 0.0351 | 0.0896 | 0.0529 | 0.0501 | 0.0532 | 8.9039 | 0.96 | 0.96 | 0.96 | 0.96 | 0.96 |
| UST2 | 2 | 1.6676 | 0.4004 | 0.5899 | 0.3916 | 0.3905 | 0.0029 | 172.5321 | 0.60 | 0.60 | 0.60 | 0.60 | 0.60 |
| UST3 | 2 | 1.5077 | 0.3367 | 0.5196 | 0.3773 | 0.3696 | 0.0205 | 23.8898 | 0.66 | 0.66 | 0.66 | 0.66 | 0.66 |
| UST4 | 2 | 1.8661 | 0.4641 | 0.6568 | 0.4644 | 0.432 | 0.0698 | 6.662 | 0.54 | 0.54 | 0.54 | 0.54 | 0.54 |
| UST5 | 2 | 1.3922 | 0.2817 | 0.4553 | 0.3302 | 0.3169 | 0.0403 | 11.9212 | 0.72 | 0.72 | 0.72 | 0.72 | 0.72 |
| UST6 | 2 | 1.9898 | 0.4974 | 0.6906 | 0.4997 | 0.4848 | 0.03 | 16.1876 | 0.50 | 0.50 | 0.50 | 0.50 | 0.50 |
| UST7 | 2 | 1.1128 | 0.1014 | 0.2089 | 0.058 | 0.0553 | 0.0458 | 10.4141 | 0.90 | 0.90 | 0.90 | 0.90 | 0.90 |
| UST8 | 2 | 1.3243 | 0.2449 | 0.4101 | 0.2867 | 0.2588 | 0.0972 | 4.6433 | 0.76 | 0.76 | 0.76 | 0.76 | 0.76 |
| UST9 | 2 | 1.1734 | 0.1478 | 0.2796 | 0.1758 | 0.1724 | 0.0195 | 25.1003 | 0.85 | 0.85 | 0.85 | 0.85 | 0.85 |
| UST10 | 2 | 1.9382 | 0.4841 | 0.6771 | 0.4738 | 0.4653 | 0.018 | 27.2969 | 0.52 | 0.52 | 0.52 | 0.52 | 0.52 |
| UST11 | 2 | 1.2582 | 0.2052 | 0.359 | 0.2395 | 0.2086 | 0.1291 | 3.3742 | 0.79 | 0.79 | 0.79 | 0.79 | 0.79 |
| UST12 | 2 | 1.9994 | 0.4998 | 0.693 | 0.4976 | 0.4856 | 0.0242 | 20.1614 | 0.50 | 0.50 | 0.50 | 0.50 | 0.50 |
| UST13 | 2 | 1.3243 | 0.2449 | 0.4101 | 0.3608 | 0.2339 | 0.3517 | 0.9218 | 0.76 | 0.76 | 0.76 | 0.76 | 0.76 |
| UST14 | 2 | 1.7935 | 0.4424 | 0.6344 | 0.4502 | 0.4421 | 0.018 | 27.2251 | 0.56 | 0.56 | 0.56 | 0.56 | 0.56 |
| UST15 | 2 | 1.2366 | 0.1913 | 0.3405 | 0.2879 | 0.1753 | 0.391 | 0.7787 | 0.81 | 0.81 | 0.81 | 0.81 | 0.81 |
| UST16 | 2 | 1.8312 | 0.4539 | 0.6463 | 0.4933 | 0.3888 | 0.2118 | 1.861 | 0.55 | 0.55 | 0.55 | 0.55 | 0.55 |
| UST17 | 2 | 1.5077 | 0.3367 | 0.5196 | 0.3972 | 0.3606 | 0.0922 | 4.9258 | 0.66 | 0.66 | 0.66 | 0.66 | 0.66 |
| UST18 | 2 | 1.6228 | 0.3838 | 0.5719 | 0.3668 | 0.3625 | 0.0116 | 42.4938 | 0.62 | 0.62 | 0.62 | 0.62 | 0.62 |
| UST19 | 2 | 1.1942 | 0.1626 | 0.3009 | 0.2037 | 0.1904 | 0.0654 | 7.1479 | 0.84 | 0.84 | 0.84 | 0.84 | 0.84 |
| UST20 | 2 | 1.8312 | 0.4539 | 0.6463 | 0.4518 | 0.4231 | 0.0636 | 7.3555 | 0.55 | 0.55 | 0.55 | 0.55 | 0.55 |
| UST21 | 2 | 1.4382 | 0.3047 | 0.4826 | 0.3283 | 0.3184 | 0.03 | 16.1645 | 0.70 | 0.70 | 0.70 | 0.70 | 0.70 |
| UST22 | 2 | 1.5077 | 0.3367 | 0.5196 | 0.3698 | 0.3579 | 0.0322 | 15.0497 | 0.66 | 0.66 | 0.66 | 0.66 | 0.66 |
| UST23 | 2 | 1.2366 | 0.1913 | 0.3405 | 0.2304 | 0.192 | 0.1667 | 2.5003 | 0.81 | 0.81 | 0.81 | 0.81 | 0.81 |
| UST24 | 2 | 1.9898 | 0.4974 | 0.6906 | 0.4999 | 0.4887 | 0.0224 | 21.8038 | 0.50 | 0.50 | 0.50 | 0.50 | 0.50 |
| UST25 | 2 | 1.9692 | 0.4922 | 0.6853 | 0.4908 | 0.4892 | 0.0033 | 151.284 | 0.51 | 0.51 | 0.51 | 0.51 | 0.51 |
| UST26 | 2 | 1.8491 | 0.4592 | 0.6518 | 0.4711 | 0.4619 | 0.0196 | 25.0551 | 0.54 | 0.54 | 0.54 | 0.54 | 0.54 |
| UST27 | 2 | 1.055 | 0.0521 | 0.1234 | 0.0578 | 0.0554 | 0.0405 | 11.8485 | 0.95 | 0.95 | 0.95 | 0.95 | 0.95 |
| UST28 | 2 | 1.9842 | 0.496 | 0.6892 | 0.4894 | 0.473 | 0.0336 | 14.3641 | 0.50 | 0.50 | 0.50 | 0.50 | 0.50 |
| UST29 | 2 | 1.7935 | 0.4424 | 0.6344 | 0.4626 | 0.4494 | 0.0285 | 17.048 | 0.56 | 0.56 | 0.56 | 0.56 | 0.56 |
| UST30 | 2 | 1.7935 | 0.4424 | 0.6344 | 0.4719 | 0.4464 | 0.0541 | 8.7408 | 0.56 | 0.56 | 0.56 | 0.56 | 0.56 |
| UST31 | 2 | 1.28 | 0.2188 | 0.3768 | 0.2913 | 0.2719 | 0.0665 | 7.0224 | 0.78 | 0.78 | 0.78 | 0.78 | 0.78 |
| UST32 | 2 | 1.9943 | 0.4986 | 0.6917 | 0.4987 | 0.4943 | 0.0089 | 55.9439 | 0.50 | 0.50 | 0.50 | 0.50 | 0.50 |
| UST33 | 2 | 1.28 | 0.2188 | 0.3768 | 0.2644 | 0.2154 | 0.1852 | 2.1997 | 0.78 | 0.78 | 0.78 | 0.78 | 0.78 |
| UST34 | 2 | 1.9382 | 0.4841 | 0.6771 | 0.4933 | 0.4661 | 0.0551 | 8.5702 | 0.52 | 0.52 | 0.52 | 0.52 | 0.52 |
| Mean |  | 1.5799± 0.3260* | 0.3383± 0.1454 | 0.5081±0.1775 | 0.3611±0.0187 | 0.3367± 0.0192 | 0.0678 | 6.8753 | 0.66 | 0.66 | 0.66 | 0.66 | 0.66 |
| n_a_ = Observed number of alleles; ne = Effective number of alleles [Kimura and Crow (1964); H = Nei's (1973); gene diversity; I = Shannon's Information index [Lewontin (1972)]; Nm = estimate of gene flow from Gst or Gcs. E.g., Nm = 0.5(1 - Gst)/Gst and calculated according to McDermott and McDonald (1993)  **The Overall Ewens-Watterson Test for Neutrality performed according to Manly (1985)  ^a^Deviations from neutrality; F values; L95 and U95 depict lower and upper confidence limits at 95% confidence. Values have been obtained on 1000 simulations. | | | | | | | | | | | | | |
