# Supplementary material for: Phylogeography and Population Structure Analysis Reveal Diversity by Gene Flow and Mutation in Ustilago segetum (Pers.) Roussel tritici Causing Loose Smut of Wheat
Source: Front Microbiol. 2019 May 15;10:1072. doi: 10.3389/fmicb.2019.01072 (PMC6529584; doi:10.3389/fmicb.2019.01072)
Supplement: Supplementary file 2 [file Data_Sheet_2.docx]

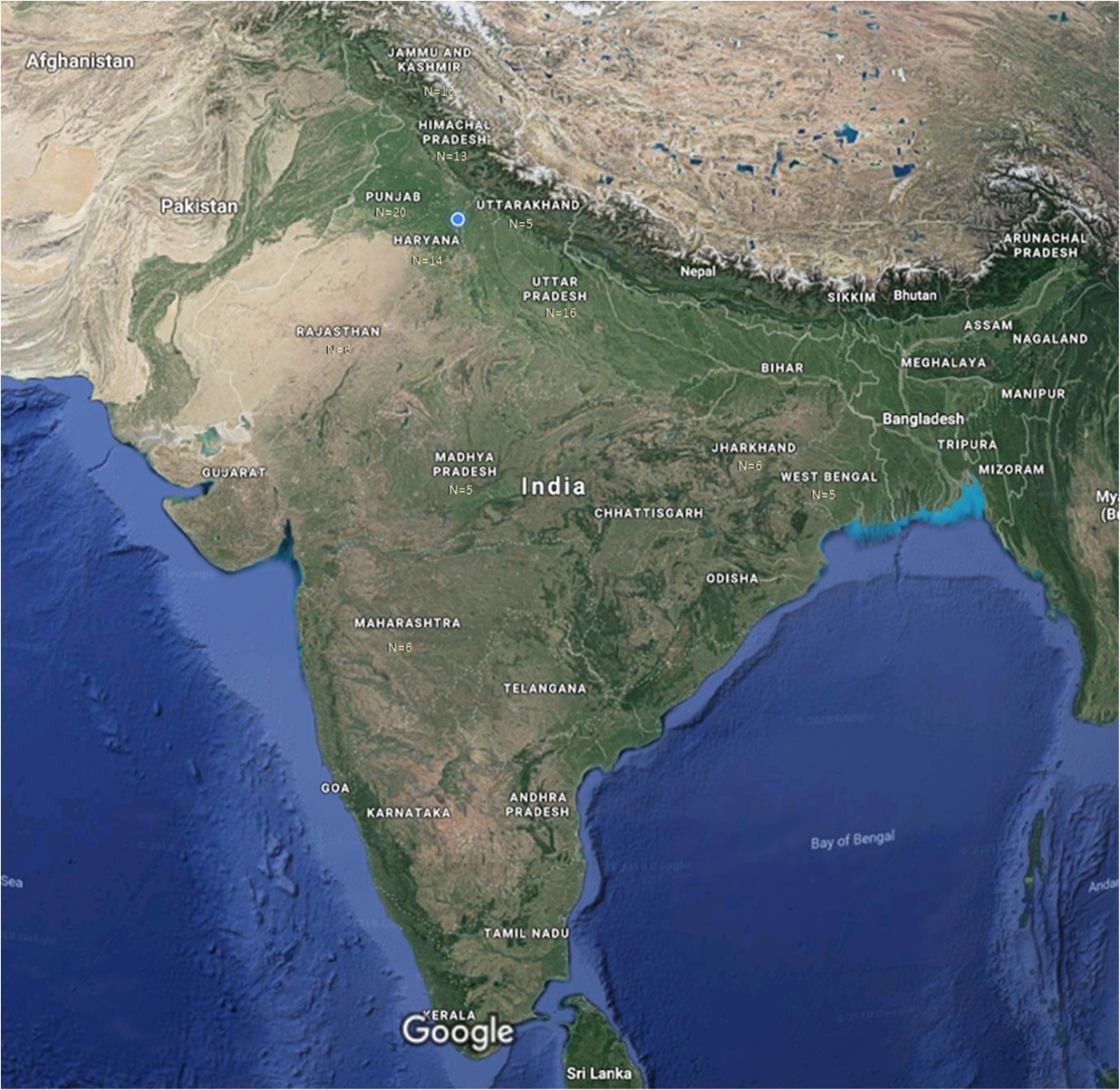


Fig S1: Geographic sites of wheat fields in India where UST isolates were sampled


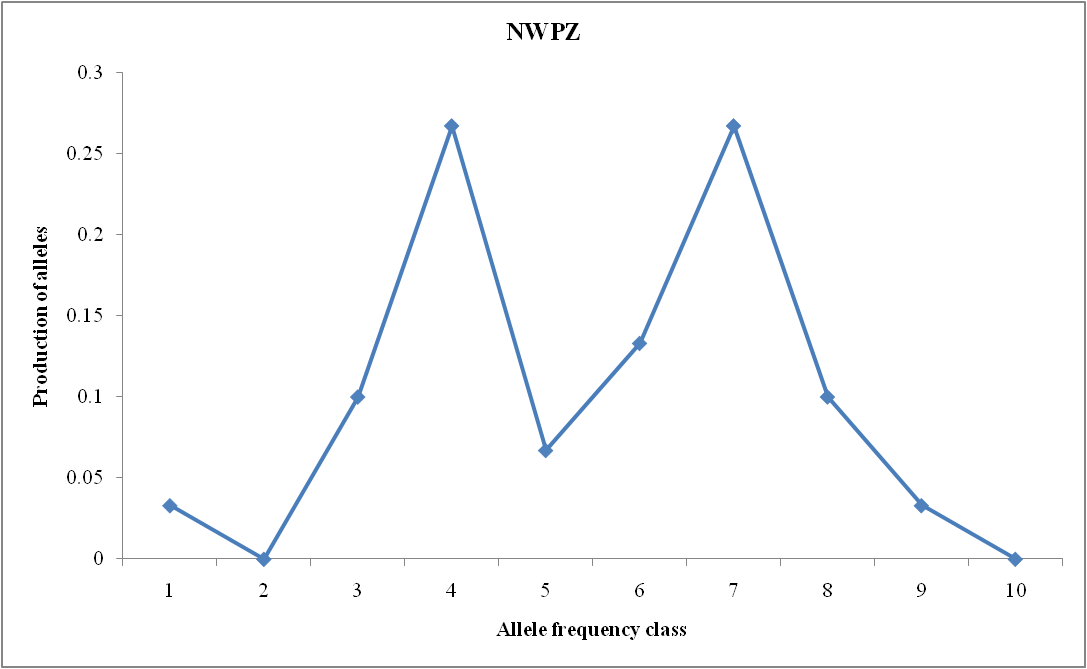


A)


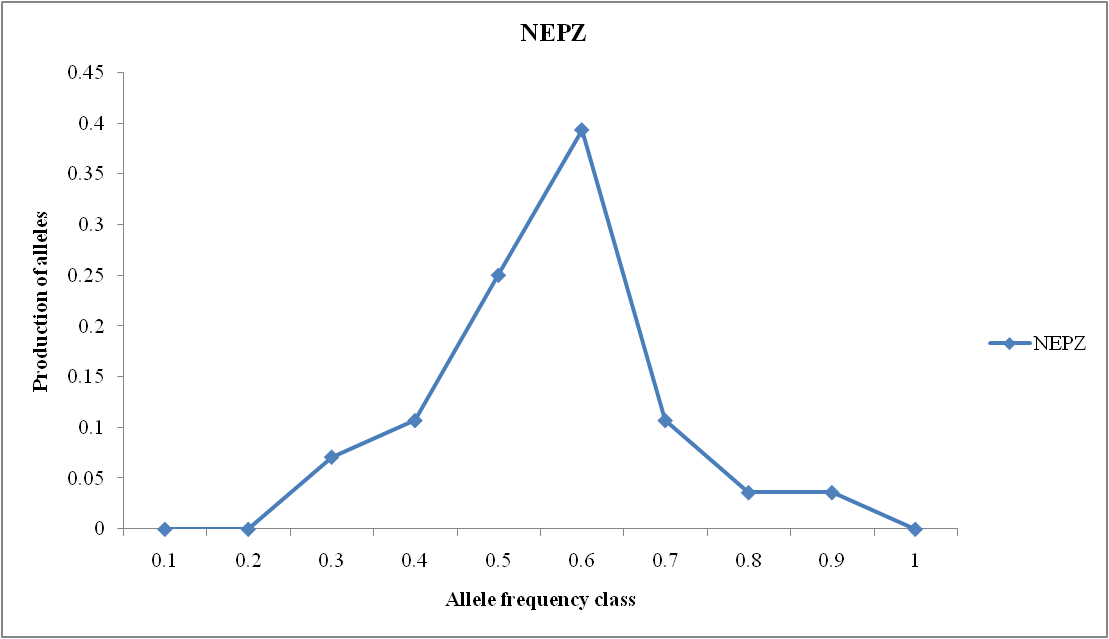


B)


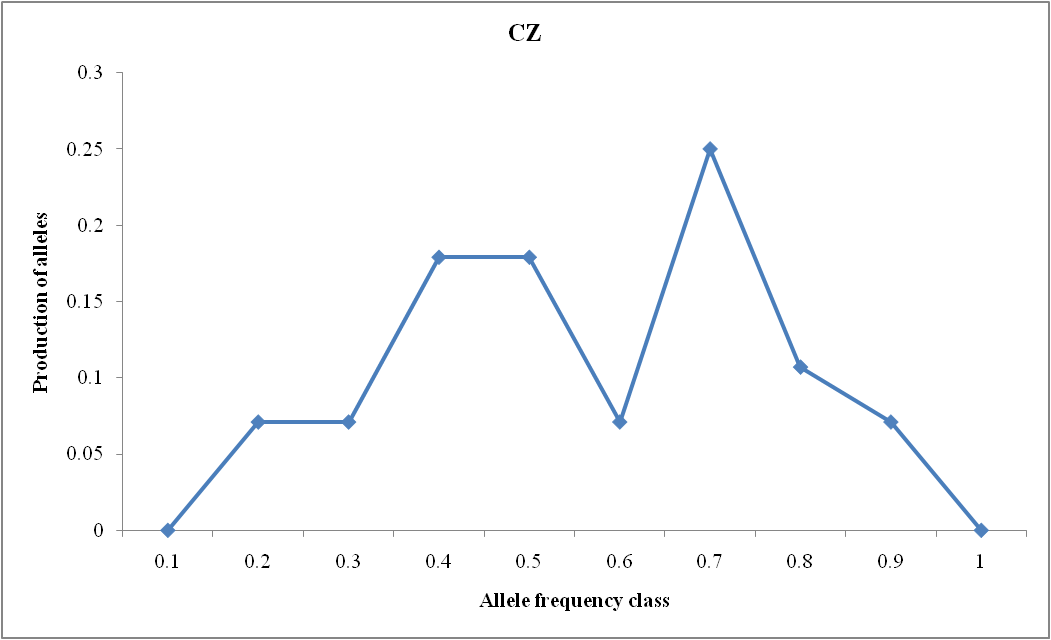


C)


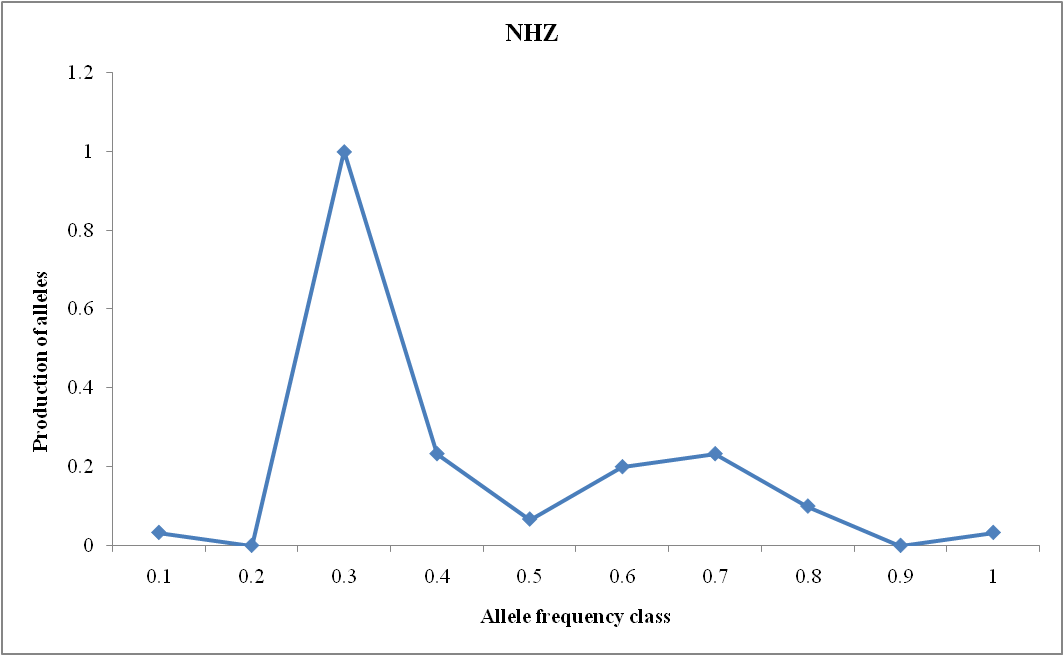


D)

**Fig S2:** L-shaped mode shift graph showing the absence of bottleneck in UST populations collected from NWPZ (A), NEPZ (B), CZ (C) and NHZ **(D)**
